# Genome Sequence of the Acidophilic Nonsulfur Purple Photosynthetic Alphaproteobacterium *Rhodovastum atsumiense,* a Divergent Member of the *Acetobacteraceae* Family

**DOI:** 10.1128/MRA.01541-19

**Published:** 2020-02-06

**Authors:** Dayana Montano Salama, Terry E. Meyer, John A. Kyndt

**Affiliations:** a College of Science and Technology, Bellevue University, Bellevue, Nebraska, USA; b Department of Chemistry and Biochemistry, The University of Arizona, Tucson, Arizona, USA; Georgia Institute of Technology

## Abstract

The genome sequence of the acidophile Rhodovastum atsumiense was determined for comparison with that of Rhodopila globiformis. Both genomes are unusually large for purple bacteria (7.10 Mb and 7.25 Mb, respectively), and they have an average nucleotide identity of 72%. This value is remarkably similar to the average nucleotide identity values for *Acidisphaera*, *Elioraea*, and *Paracraurococcus*, all aerobic anoxygenic phototrophs.

## ANNOUNCEMENT

Acidophilic purple photosynthetic bacteria are relatively unusual. There are two groups, which are only distantly related, i.e., those in the *Bradyrhizobiaceae* family, including Rhodoblastus acidophilus (1) and Rhodoblastus sphagnicola (2), and those in the *Acetobacteraceae* family, including Rhodopila globiformis (3) and Rhodovastum atsumiense (4). One of the most important characteristics of these bacteria is the optimal growth pH, which is 4.8 to 5.0 for R. globiformis and 6.0 to 6.5 for R. atsumiense (3, 4). The redox potentials of cytochrome *c*_2_ and high-potential iron-sulfur protein (HiPIP) are surprisingly high, in the region of 400 mV (5–7), but the reason for this observation remains unknown. If nothing else, it suggests that R. globiformis lives in a highly aerobic environment. Another observation is that cells die immediately if they are overgrown, which is generally not the case with other purple bacteria (8, 9). Acidisphaera rubrifaciens is an aerobic anoxygenic phototroph (AAP), whose rRNA is relatively closely related to that of R. globiformis (94.4% identity) and R. atsumiense (95.5% identity) (10). The genome sequence of R. globiformis was determined previously (11), and we now report the genome sequence of R. atsumiense.

Rhodovastum atsumiense was originally isolated from submerged paddy soil from the Atsumi Peninsula in Japan (4). Genomic DNA of R. atsumiense (strain DSM 21279) was obtained from DSMZ. DNA analysis using Qubit and NanoDrop instruments showed an *A*_260_/*A*_280_ ratio of 1.79. The sequencing library was prepared using the Illumina Nextera DNA Flex library preparation kit. The genome was sequenced with an Illumina MiniSeq system using 500 μl of a 1.8 pM library. Paired-end (2 × 150-bp) sequencing generated 2,592,590 reads and 202.3 Mbp (35× coverage). Quality control of the reads was performed using FastQC within BaseSpace (version 1.0.0; Illumina), using a k-mer size of 5 and contamination filtering. We assembled the genome *de novo* using SPAdes (version 3.10.0) (12) through PATRIC (13). This assembly yielded 226 contigs (>300 bp), with the largest being 264,348 bp; the *N*_50_ was 104,226 bp. The genome had a GC content of 68.7% and was 7,097,890 bp long, which is larger than the average size of purple bacterial genomes (2.5 to 5.5 Mbp) (14) but similar to the size of the Rhodopila globiformis genome (7.25 Mb) (11). The genome was annotated using the RAST tool kit (15) within PATRIC (13), and this showed our strain to have 6,942 coding sequences and 50 tRNAs.

**FIG 1:**
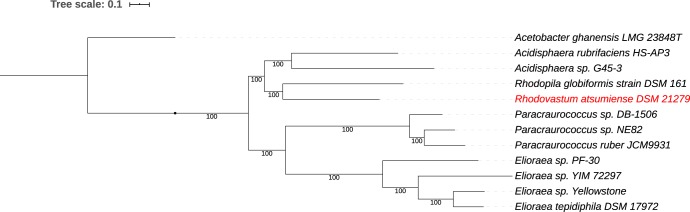
Phylogenetic tree for the whole genome of Rhodovastum atsumiense, in comparison to its closest relatives. The phylogenetic tree was generated using the codon tree method within PATRIC (13), which used cross-genus families (PGFams) as homology groups; 385 PGFams were found among these selected genomes using Codon Tree analysis, and the aligned proteins and coding DNA from single-copy genes were used for RAxML analysis (17, 18). Acetobacter ghanensis was used as an outgroup. iTOL was used for the tree visualization.

A JSpeciesWS comparison (16) of the average nucleotide identity (ANI) of the *Rhodovastum atsumiense* genome indicated 72.0% identity to the R. globiformis genome and 72.5% to Acidisphaera rubrifaciens DSM 16009. Other AAPs, namely, Elioraea tepidiphila DSM 17972 and Paracraurococcus ruber, both demonstrated ANI values of 70.5%. The ANI values for Rhodovastum atsumiense are clearly below the proposed 95% cutoff value for genome definition of a species (16). ANI analysis also showed that R. atsumiense and the AAPs mentioned above are not very closely related (<72% identity). Phylogenetic analysis of the R. atsumiense genome using RAxML within PATRIC (17, 18) showed R. globiformis as the closest relative, followed by *Acidisphaera* and, more distantly, *Paracraurococcus* and *Elioraea* (Fig. 1).

R. atsumiense has both cytochrome *c*_2_ and HiPIP, and the HiPIP gene is located in the same place as in R. globiformis, downstream of PuhA and the cytochromes *c*_2_ that donate electrons to the photosynthetic reaction center in other species of purple bacteria. This finding suggests that HiPIP, rather than cytochrome *c*_2_, is the electron donor in these two species, although the proteins could react interchangeably.

### Data availability.

This whole-genome shotgun project has been deposited in DDBJ/ENA/GenBank under accession number VWPK00000000; the version described in this paper is version VWPK01000000. The raw sequencing reads have been submitted to SRA under accession number SRR10679485.
